# Nomogram prediction for the risk of venous thromboembolism in patients with lung cancer

**DOI:** 10.1186/s12935-023-02882-1

**Published:** 2023-03-05

**Authors:** Haike Lei, Dan Tao, Ningning Zhang, Mao Sun, Lisi Sun, Dingyi Yang, Yong Jiang, Wei Zhou, Yue Xie, Ying Wang

**Affiliations:** 1 Chongqing Cancer Multi-omics Big Data Application Engineering Research Center, Chongqing University Cancer Hospital, Chongqing, China; 2 Department of Radiation Oncology, Chongqing University Cancer Hospital, 181, Hanyu Road, Shapingba District, Chongqing, 400030 China; 3 Department of Breast Cancer Center, Chongqing University Cancer Hospital, Chongqing, China; 4 Chongqing Key Laboratory of Translational Research for Cancer Metastasis and Individualized Treatment, Chongqing University Cancer Hospital, Chongqing, China

**Keywords:** Venous thromboembolism, Nomogram, Lung cancer, Predictive model

## Abstract

**Objective:**

The aim of this study was to establish a nomogram graph model to accurately predict the venous thromboembolism (VTE) risk probability in the general population with lung cancer.

**Methods:**

Based on data from patients with lung cancer in Chongqing University Cancer Hospital of China, the independent risk factors of VTE were identified by the logistic univariable and multivariable analysis and were integrated to construct a nomogram, which was validated internally. The predictive effectiveness of the nomogram was evaluated by the receiver operating characteristic curve (ROC) and calibration curve.

**Results:**

A total of 3398 lung cancer patients were included for analysis. The nomogram incorporated eleven independent VTE risk factors including karnofsky performance scale (KPS), stage of cancer, varicosity, chronic obstructive pulmonary disease (COPD), central venous catheter (CVC), albumin, prothrombin time (PT), leukocyte counts, epidermal growth factor receptor tyrosine kinase inhibitor (EGFR-TKI), dexamethasone, and bevacizumab. The C-index of the nomogram model was 0.843 and 0.791 in the training and validation cohort, respectively, demonstrating good discriminative power. The calibration plots of the nomogram revealed excellent agreement between the predicted and actual probabilities.

**Conclusions:**

We established and validated a novel nomogram for predicting the risk of VTE in patients with lung cancer. The nomogram model could precisely estimate the VTE risk of individual lung cancer patients and identify high-risk patients who are in need of a specific anticoagulation treatment strategy.

## Introduction

Venous thromboembolism (VTE) mainly comprises deep vein thrombosis (DVT) and pulmonary embolism (PE), which is one of the most common complications of malignant tumors [1]. Numerous previous studies have found that VTE in cancer patients was associated with increased mortality and worse quality of life [2]. Lung cancer is the second most prevalent cancer and the leading cause of tumor-related death globally [3]. Several studies have revealed that lung cancer patients are more prone to developing VTE [4, 5]. Zhang et al. [5] reported that VTE events occurred in up to 13.2% of patients with newly diagnosed lung cancer. In recent years, target therapy and immunotherapy dramatically improved the survival of lung cancer patients [6, 7]. However, these advancements in systematic treatment and prolonged survival increased the VTE incidence in lung cancer patients [8–10].

Several models have been developed to predict the VTE risk in patients, including Caprini score [11, 12], Padua score [13], Rogers score [14], and Khorana score [15]. These models were widely applied in clinical practice and accurately identified patients with a high risk of VTE. However, these models were designed for different subgroups of populations. Caprini model was mainly developed to evaluate the VTE risk of patients undergoing lung cancer resections [12]. In contrast, the Padua risk model was used for predicting VTE risk in hospitalized medical patients [13]. Lung cancer patients are a particular subgroup with unique characteristics, such as the driver gene status and treatment regimens. The application of these models to predict the VTE risk of lung cancer patients has significant limitations, for they can’t include all VTE risk factors in lung cancer patients. Thus, there is an urgent need to develop a VTE prediction model specifying lung cancer patients to guide clinical practice.

The Nomogram model is a visual graphic tool for accurately predicting each patient's risk probability of clinical events [16]. By producing a user-friendly graph, this prediction approach converts the conventional regression model into a visual risk assessment for each patient, which is undeniably practical and accurate. This model has been proven to be an accurate method for predicting cancer prognosis. Nomograms for VTE risk assessment have been studied in various cancers, including breast cancer [17], ovarian cancer [18, 19], lymphoma [20], and spinal metastasis tumors [21]. Recently, two studies [22, 23] developed a nomogram model to predict the risk probability of postoperative VTE in patients with early-stage lung cancer. However, in the era of precision medicine, there is still a lack of a nomogram model predicting VTE risk for the general population with lung cancer.

Therefore, in the current study, we construct a nomogram graph model to accurately predict the VTE risk probability in the general population with lung cancer. With the use of this model, physicians may precisely identify patients who are at high risk for developing VTE and can implement early preventive and treatment strategies to lower the chance of developing thrombosis.

## Materials and methods

### Patient population

Data were collected from the patients’ medical records in Chongqing University Cancer Hospital of China. As our previous reports [24], the inclusion criteria include: (1) age ≥ 18 years; (2) at least once hospitalization; (3) with newly histologically confirmed lung carcinoma; (4) with hospitalization date from January 2013 to December 2019. The exclusive criteria were as follows: (1) VTE occurred before the diagnosis of lung cancer and (2) died within 48 h after admission; (3) patients with incomplete information. All available data on the database were used to maximize the power and generalizability of the results. Patients who underwent several hospital stays were only counted once in the analysis.

### Construction of the nomogram and statistical analysis

For nomogram construction and validation, we randomly divided all the patients into training (n = 2379) and validation (n = 1019) cohorts in a ratio of 7:3. In the training cohort, the association between clinical variables and VTE was analyzed using univariate logistic regression analysis. Variables that achieved significance at *P* < 0.05 in univariate analysis and variables with obvious clinical significance were entered into the multivariable analyses via Logistic regression model. Statistical analyses to identify independent predictive factors for VTE were performed by multivariable analysis. Based on the results of the multivariable analysis, a nomogram was constructed.

The nomogram was validated internally in the training cohort and externally in the validation cohort. The Harrell’s C-index (the concordance statistic) and the area under the receiver operating characteristic curve (AUC) were used to evaluate the discriminative ability of the nomogram. Bootstrapping method with 1000 resamples was utilized to generate the calibration curves for validation of the nomogram in the training cohort and in the validation cohort. The scores of each variable were calculated using the “nomogramEx” package in R. Based on the scores of each variable, the total scores for each patient could be calculated. The decision curve analysis (DCA) of the nomogram was plotted using the "rmda" package.

All analyses were performed with R 4.1.2 (http://www.r-project.org). Statistical significance was defined as a two-sided p value of < 0.05.

## Results

### Clinicopathologic characteristics of patients

**Fig. 1:**
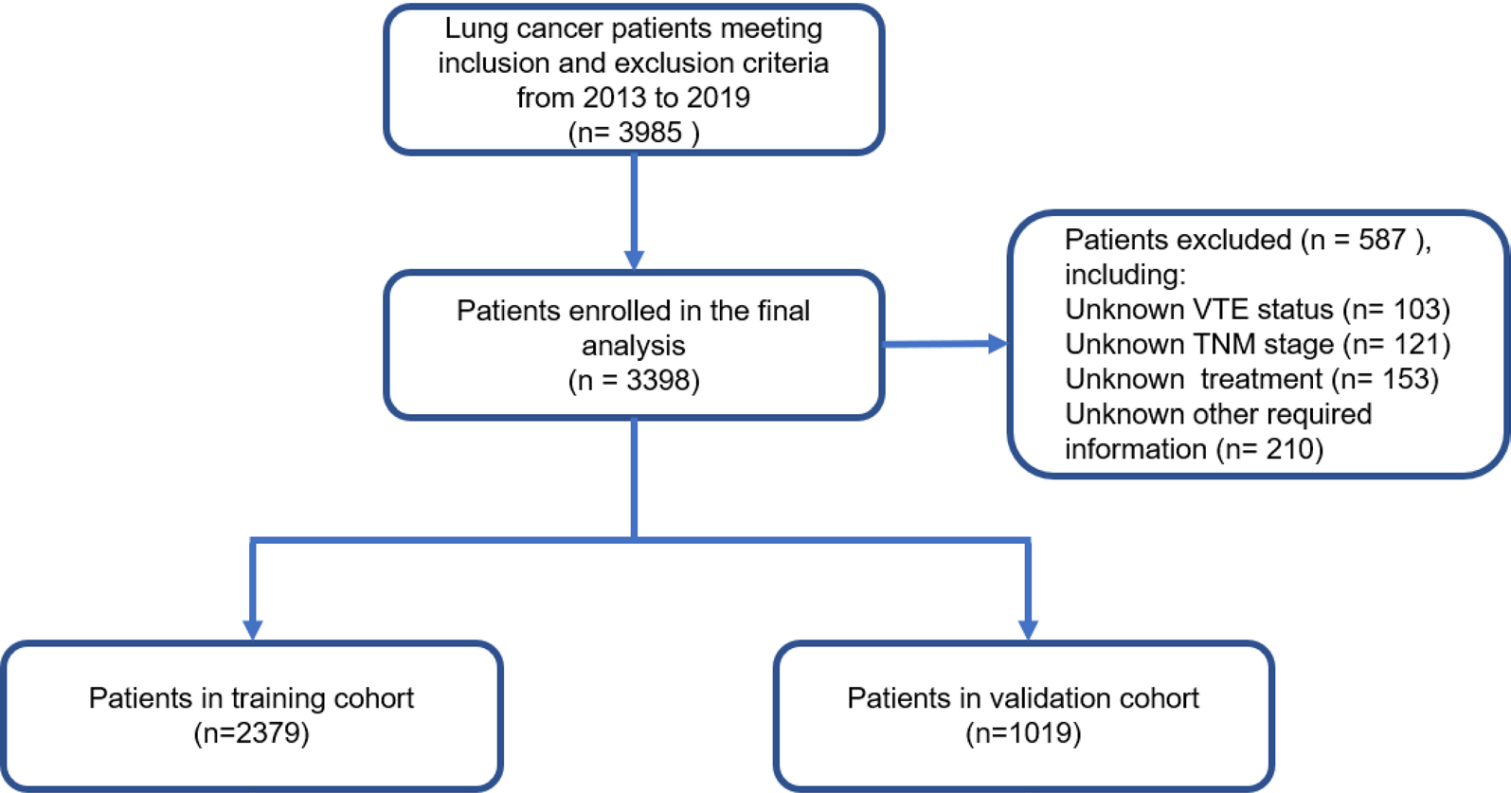
Flowchart detailing the inclusion and exclusion criteria that resulted in the final study cohorts

A total of 3985 lung cancer patients from 2013 to 2019 were screened for this study (Fig. 1). Finally, 3398 patients were enrolled in the analysis. The eligible patients were divided into training cohort (n = 2379) and validation cohorts (n = 1019) according to the random split-sample method (split ratio: 7:3). In the training cohort, 89 (3.7%) patients experienced VTE during their hospital stay. While in the validation cohort, 36 (3.5%) patients experienced VTE. The baseline clinicopathologic characteristics of the patients and the association between VTE and clinicopathological features are shown in Table 1.

**Table 1:** Demographics and clinicopathologic characteristics of the training and validation sets

Variables	Total	Training set	Validation set	*P-value*
	N = 3398	N = 2379	N = 1019	
Age (mean (SD))	64.028 (10.307)	64.055 (10.211)	63.966 (10.533)	0.817
Sex				
Female	1024 (30.14)	709 (29.80)	315 (30.91)	0.545
Male	2374 (69.86)	1670 (70.20)	704 (69.09)	
KPS (mean (SD))	76.758 (11.01)	76.598 (11.38)	77.130 (10.07)	0.197
Stage of cancer				
I–II	424 (12.48)	306 (12.86)	118 (11.58)	0.582
III	749 (22.04)	523 (21.98)	226 (22.18)	
IV	2225 (65.48)	1550 (65.15)	675 (66.24)	
Pathological type				
NSCLC	2973 (87.49)	2081 (87.47)	892 (87.54)	1
SCLC	425 (12.51)	298 (12.53)	127 (12.46)	
A history of VTE				
No	3226 (94.94)	2254 (94.75)	972 (95.39)	0.486
Yes	172 (5.06)	125 (5.25)	47 (4.61)	
Varicosity				
No	3377 (99.38)	2366 (99.45)	1011 (99.21)	0.566
Yes	21 (0.62)	13 (0.55)	8 (0.79)	
COPD				
No	2732 (80.40)	1906 (80.12)	826 (81.06)	0.557
Yes	666 (19.60)	473 (19.88)	193 (18.94)	
CVC				
No	3288 (96.76)	2307 (96.97)	981 (96.27)	0.340
Yes	110 (3.24)	72 (3.03)	38 (3.73)	
History of malignant tumor				
No	3349 (98.56)	2345 (98.57)	1004 (98.53)	1
Yes	49 (1.44)	34 (1.43)	15 (1.47)	
BMI				
< 18.5	296 (8.71)	209 (8.79)	87 (8.54)	0.942
18.5–23.9	1870 (55.03)	1305 (54.85)	565 (55.45)	
≥ 24	1232 (36.26)	865 (36.36)	367 (36.02)	
PLT (mean (SD))	222.456 (99.741)	222.434 (99.599)	222.509 (100.121)	0.984
Albumin (mean (SD))	38.844 (6.249)	38.746 (6.318)	39.071 (6.083)	0.166
D.dimer (mean (SD))	2.081 (3.641)	2.073 (3.676)	2.099 (3.558)	0.848
PT (mean (SD))	11.978 (1.925)	11.966 (1.800)	12.005 (2.190)	0.587
APTT (mean (SD))	28.438 (5.515)	28.563 (5.763)	28.146 (4.877)	0.044
Hemoglobin (mean (SD))	121.793 (20.515)	121.877 (20.666)	121.597 (20.166)	0.716
Leukocyte (mean (SD))	5.541 (5.344)	5.624 (5.342)	5.349 (5.345)	0.169
Creatinine (mean (SD))	66.794 (37.724)	66.658 (36.201)	67.112 (41.076)	0.748
Mitomycin				
No	3390 (99.76)	2374 (99.79)	1016 (99.71)	0.938
Yes	8 (0.24)	5 (0.21)	3 (0.29)	
rh-Endostatin				
No	3290 (96.82)	2309 (97.06)	981 (96.27)	0.275
Yes	108 (3.18)	70 (2.94)	38 (3.73)	
EGFR.TKI				
No	2995 (88.14)	2089 (87.81)	906 (88.91)	0.395
Yes	403 (11.86)	290 (12.19)	113 (11.09)	
Dexamethasone				
No	882 (25.96)	619 (26.02)	263 (25.81)	0.932
Yes	2516 (74.04)	1760 (73.98)	756 (74.19)	
Platinum				
No	2751 (80.96)	1929 (81.08)	822 (80.67)	0.813
Yes	647 (19.04)	450 (18.92)	197 (19.33)	
Bevacizumab				
No	3320 (97.70)	2322 (97.60)	998 (97.94)	0.636
Yes	78 (2.30)	57 (2.40)	21 (2.06)	

SD: standard deviation; KPS: karnofsky performance scale; NSCLC: non-small cell lung cancer; SCLC: small cell lung cancer; VTE: venous thromboembolism; COPD: chronic obstructive pulmonary disease; CVC: central venous catheter; BMI: body mass index; PLT: platelet; PT: prothrombin time; APTT: activated partial thromboplastin time; EGFR-TKI: epidermal growth factor receptor tyrosine kinase inhibitor

### Independent predictive factors in the training set

The results of the logistic univariable analysis are shown in Table 2. Male gender (male vs. female; *P* = 0.004) was an unfavorable factor for VTE. Stage IV (I–II vs. III vs. IV; *P* = 0.004) and NSCLC type (NSCLC vs. SCLC; *P* = 0.027) were significantly associated with a higher rate of VTE. Patients with comorbidity of varicosity (*P* < 0.001) and COPD (*P* = 0.006) experienced a higher risk of VTE. In accordance with our expectation, patients with CVC showed a higher risk of VTE compared with those without CVC (*P* < 0.001). Lower albumin level was associated with a higher risk of VTE (*P* = 0.006). Patients with shorter PT have a higher risk for VTE than those with longer PT (*P* = 0.002). While we found that higher Leukocyte levels showed a higher risk of VTE (*P* = 0.01). With respect to the treatment factors which may impact the occurrence of VTE, the results revealed that EGFR-TKI (*P* < 0.001), Dexamethasone (*P* < 0.001), Platinum (*P* = 0.002), and Bevacizumab (*P* < 0.001) were the significantly favorable factors for VTE. All significant variables in the univariable analysis and variables with obvious clinical significance were entered into the multivariate logistic regression analysis using a stepwise method. All KPS (*P* = 0.048), stage of cancer (*P* = 0.015), varicosity (*P* < 0.001), COPD (*P* = 0.002), albumin (*P* = 0.006), PT (*P* = 0.015), Leukocyte (*P* = 0.006), EGFR-TKI (*P* < 0.001), Dexamethasone (*P* = 0.005), and Bevacizumab (*P* = 0.003) remained independent predictive factors in the multivariate logistic regression model.

**Table 2:** Logistic regression analysis of the risk factors for VTE in the training set

Characteristics	NO VTE (N = 2290)	VTE (N = 89)	OR (univariable)	OR (multivariable)
Age (Mean ± SD)	64.1 ± 10.2	63.4 ± 10.3	0.99 (0.97–1.01, p = .568)	
Sex				
Female	670 (29.3%)	39 (43.8%)		
Male	1620 (70.7%)	50 (56.2%)	0.53 (0.35–0.81, p = .004)	
KPS (Mean ± SD)	76.5 ± 11.5	78.3 ± 9.0	1.01 (0.99–1.04, p = .160)	1.02 (1.00–1.05, p = .048)
Stage of cancer				
I–II	304 (13.3%)	2 (2.2%)		
III	515 (22.5%)	8 (9%)	2.36 (0.50–11.19, p = .279)	2.38 (0.48–11.73, p = .287)
IV	1471 (64.2%)	79 (88.8%)	8.16 (2.00–33.39, p = .004)	6.23 (1.43–27.25, p = .015)
Pathological type				
NSCLC	1996 (87.2%)	85 (95.5%)		
SCLC	294 (12.8%)	4 (4.5%)	0.32 (0.12–0.88, p = .027)	
Varicosity				
No	2282 (99.7%)	84 (94.4%)		
Yes	8 (0.3%)	5 (5.6%)	16.98 (5.44–53.00, p < .001)	18.68 (4.10–85.10, p < .001)
COPD				
No	1845 (80.6%)	61 (68.5%)		
Yes	445 (19.4%)	28 (31.5%)	1.90 (1.20–3.01, p = .006)	2.26 (1.36–3.76, p = .002)
CVC				
No	2227 (97.2%)	80 (89.9%)		
Yes	63 (2.8%)	9 (10.1%)	3.98 (1.91–8.28, p < .001)	2.28 (0.99–5.25, p = .053)
History of malignant tumor				
No	2256 (98.5%)	89 (100%)		
Yes	34 (1.5%)	0 (0%)	0.00 (0.00-Inf, p = .974)	
BMI				
< 18.5	203 (8.9%)	6 (6.7%)		
18.5–23.9	1266 (55.3%)	39 (43.8%)	1.04 (0.44–2.49, p = .926)	0.82 (0.32–2.06, p = .666)
≥ 24	821 (35.9%)	44 (49.4%)	1.81 (0.76–4.31, p = .178)	1.39 (0.55–3.53, p = .492)
PLT (Mean ± SD)	222.9 ± 100.0	211.3 ± 87.0	1.00 (1.00–1.00, p = .283)	
Albumin (Mean ± SD)	38.8 ± 6.3	37.6 ± 6.9	0.97 (0.94–1.00, p = .078)	0.95 (0.91–0.98, p = .006)
D dimer (Mean ± SD)	2.1 ± 3.7	2.6 ± 4.0	1.03 (0.99–1.08, p = .186)	
PT (Mean ± SD)	12.0 ± 1.8	11.5 ± 2.1	0.76 (0.64–0.91, p = .002)	0.79 (0.65–0.95, p = .015)
APTT (Mean ± SD)	28.6 ± 5.8	27.5 ± 5.5	0.96 (0.92–1.00, p = .080)	
Hemoglobin (Mean ± SD)	122.0 ± 20.7	117.7 ± 19.5	0.99 (0.98–1.00, p = .052)	
Leukocyte (Mean ± SD)	5.6 ± 5.3	7.1 ± 6.3	1.04 (1.01–1.08, p = .010)	1.05 (1.01–1.09, p = .006)
Creatinine (Mean ± SD)	66.8 ± 36.7	61.7 ± 20.2	0.99 (0.98–1.00, p = .120)	
Mitomycin				
No	2285 (99.8%)	89 (100%)		
Yes	5 (0.2%)	0 (0%)	0.00 (0.00-Inf, p = .985)	
rh-Endostatin				
No	2223 (97.1%)	86 (96.6%)		
Yes	67 (2.9%)	3 (3.4%)	1.16 (0.36–3.75, p = .808)	
EGFR-TKI				
No	2044 (89.3%)	45 (50.6%)		
Yes	246 (10.7%)	44 (49.4%)	8.12 (5.25–12.57, p < .001)	4.71 (2.90–7.67, p < .001)
Dexamethasone				
No	614 (26.8%)	5 (5.6%)		
Yes	1676 (73.2%)	84 (94.4%)	6.15 (2.49–15.24, p < .001)	3.88 (1.52–9.95, p = .005)
Platinum				
No	1868 (81.6%)	61 (68.5%)		
Yes	422 (18.4%)	28 (31.5%)	2.03 (1.28–3.22, p = .002)	
Bevacizumab				
No	2247 (98.1%)	75 (84.3%)		
Yes	43 (1.9%)	14 (15.7%)	9.75 (5.12–18.60, p < .001)	3.14 (1.46–6.77, p = .003)

### Predictive nomogram for VTE

**Fig. 2:**
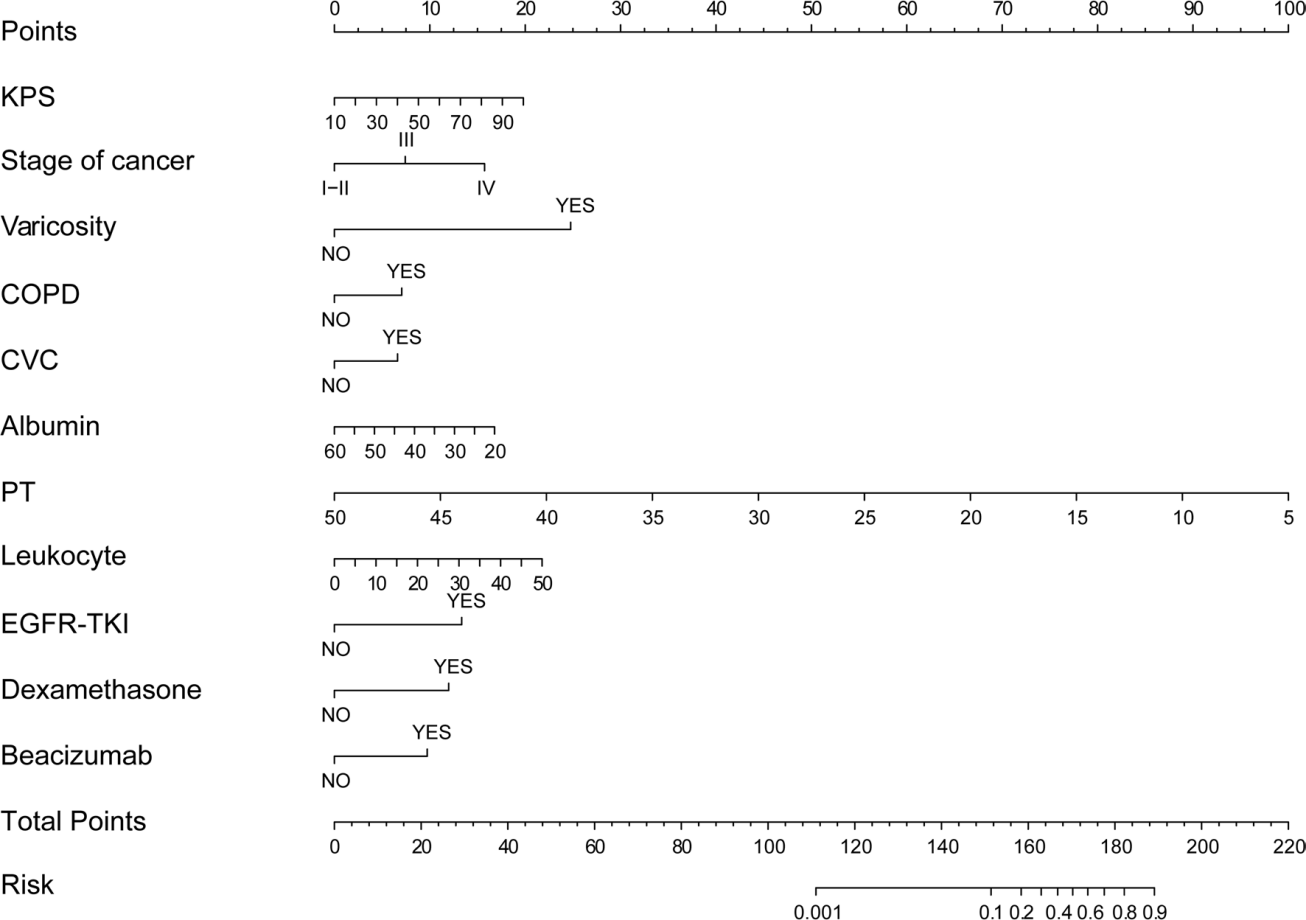
A nomogram model was constructed based on the independent risk factors identified by multivariate logistic regression analysis. The nomogram is used to find the position of each variable on the corresponding axis. Firstly, you draw a vertical line for each of the variables of your patient to the Points axis for the score of each variable; secondly, you sum up the scores of all valuables you read on the Points scale to obtain Total Points; finally, you draw a vertical line from the Total Points axis to determine the risk of VTE at the lower line of the nomogram

A nomogram that incorporated the significant predictive factors and factors with clinical significance was constructed (Fig. 2). The nomogram demonstrated that PT was the most considerable contribution to the prediction for VTE, followed by varicosity and leukocyte. KPS, stage of cancer, and albumin showed a moderate impact on VTE. Each of these variables was assigned a score on the point scale. Then, by adding up the total score and locating it on the total point scale, we were able to draw a straight line down to determine the estimated probability of VTE.

Furthermore, for clinically convenient prediction of VTE risk in lung cancer patients, an online application was developed by utilizing the R package "DynNom" (https://cran.r-project.org/web/packages/DynNom/index.html). It is available at https://cqchlungcancervte.shinyapps.io/DynNomapp/. After determining the parameters, the probability of VTE can be generated by clicking the “predict” button.

### Validation and calibration of the nomogram

**Fig. 3:**
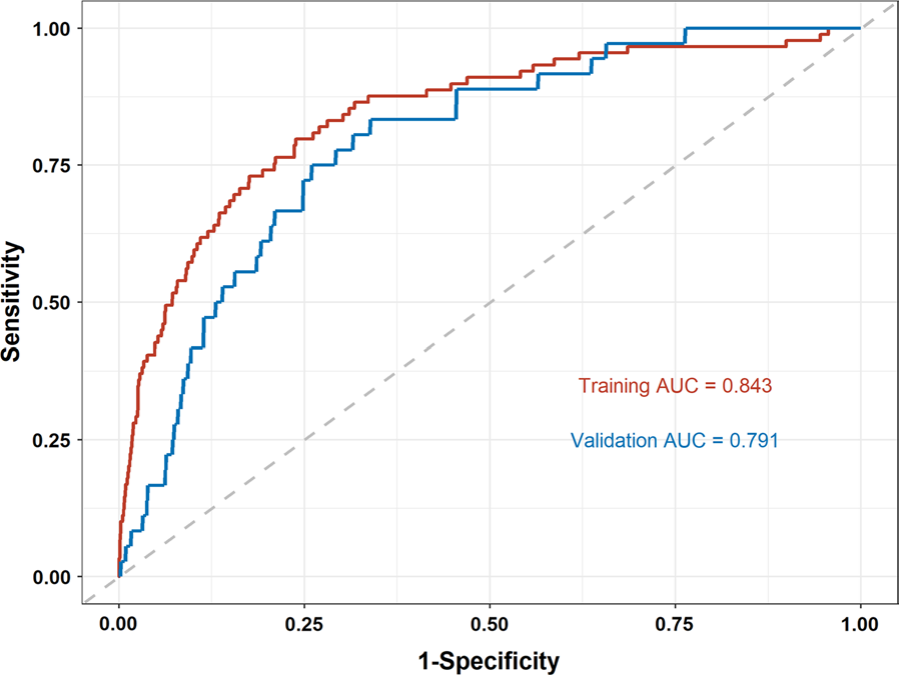
ROC curves of the nomogram for VTE risk prediction in the training and validation cohorts

**Fig. 4:**
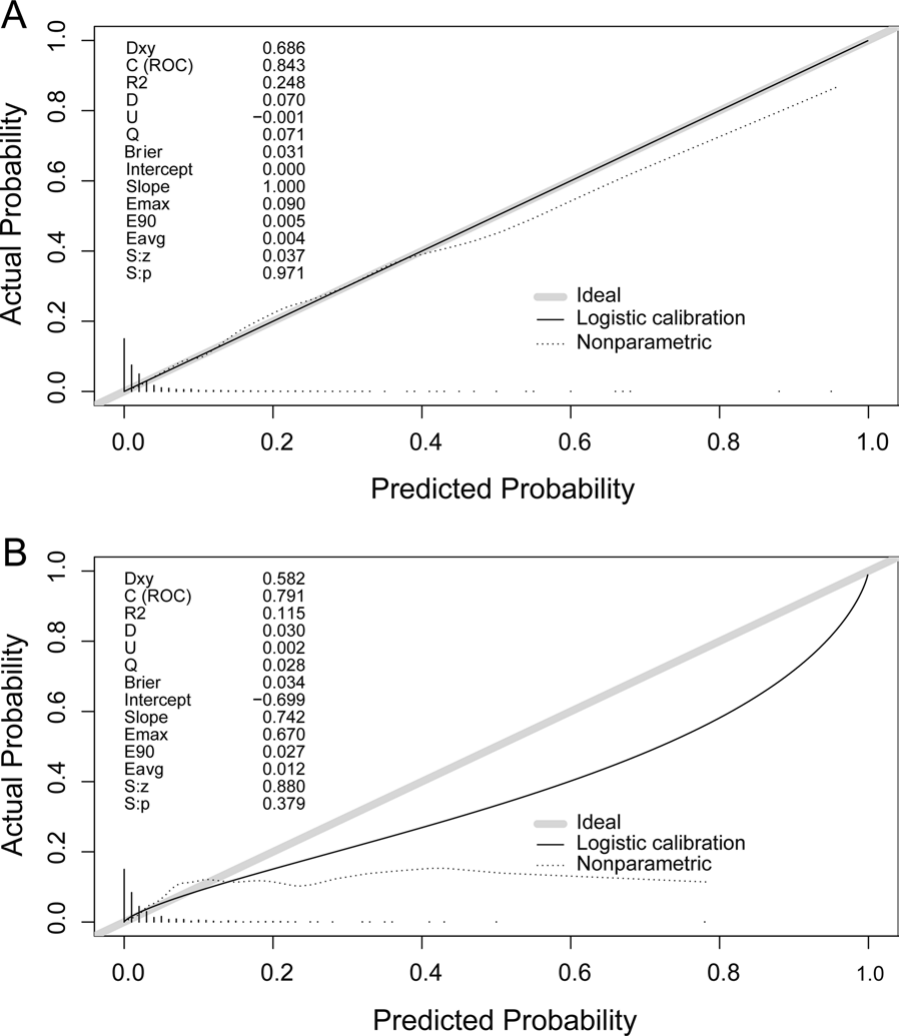
Calibration plot of the nomogram for VTE risk in the training cohort (**A**) and validation cohort (**B**)

In the training cohort, the C-index for the established nomogram to predict VTE was 0.843 with 95% CI 0.798–0.889, while in the validation cohort, the C-index was 0.791 with 95% CI 0.725–0.858 (Fig. 3). The calibration plots of the nomogram revealed that the agreement between the predicted and observed VTE risk was optimal both in training (Fig. 4A) and validation cohorts (Fig. 4B).

### Performance of the nomogram in stratifying risk of patients

**Fig. 5:**
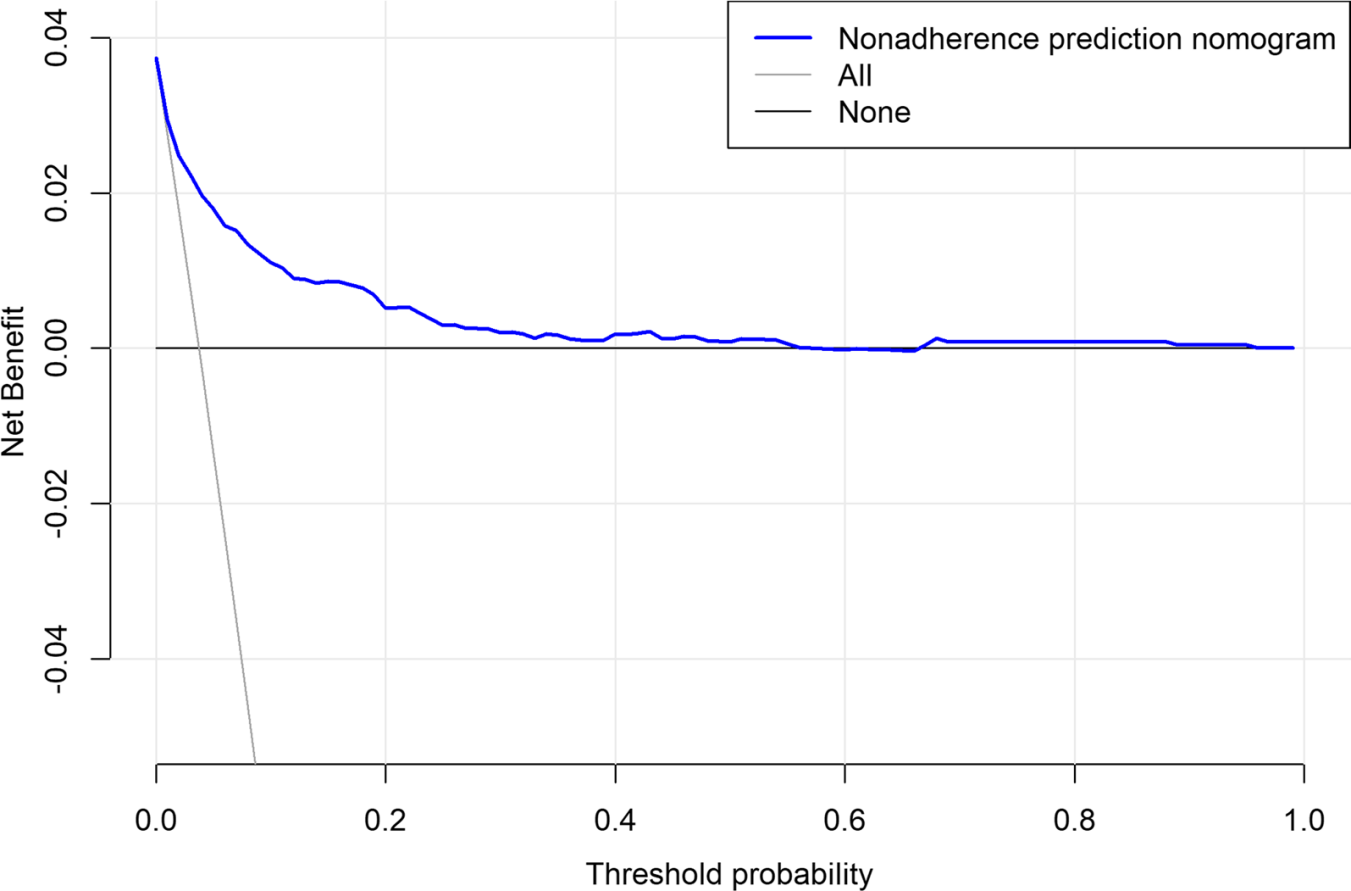
Decision-curve analysis (DCA) of VTE

DCA was performed to evaluate the clinical utility of the nomogram model by calculating its net benefit in various risk thresholds for screening. The DCA visually illustrated the model's clinical utility based on a continuum of VTE risk thresholds (X axis) and the net benefit of employing the model to stratify the risk of the patients (Y axis) relative to the hypothesis that no patient will have a VTE. The decision curve in Fig. 5 indicated that when the threshold probability for a patient or a doctor is within a range from 8 to 60%, there will be more net benefit than either treating all patients or treating none by using the nomogram to decide whether or not to conduct treatment. The decision curve revealed that the nomogram performed well and was feasible to make beneficial clinical decisions.

## Discussion

VTE is a common complication in lung cancer patients, and its occurrence significantly increases patient mortality [4]. Although assessment models, such as Caprini RAM and Padua Prediction Score, were developed to evaluate the risk of VTE [25]. However, there is few VTE risk model specifying lung cancer, the second most common cancer worldwide [3]. This study identified the dependent VTE risk factors and established a reliable nomogram to accurately predict the VTE risk for lung cancer patients. To our best knowledge, this is the first study with a large sample size that develop a nomogram for predicting the risk of VTE in the general population with stage I-IV lung cancer.

Nomogram is a method to accurately predict the probability of occurrence of each individual outcome event, which can convert a complex regression equation into a simple and visual graph [26], making the prediction model more readable and practical. This outstanding feature has led to increased interest in medical research and clinical practice for the diagram, which is frequently used to predict the prognosis of patients with cancers [16]. In this study, we constructed a nomogram prediction model for VTE risk including KPS, stage of cancer, varicosity, COPD, CVC, albumin, PT, leukocyte counts, EGFR-TKI, dexamethasone, and bevacizumab. The C-index of 0.843 demonstrates the accurate prediction power of this nomogram prediction model, which has been verified internally. Recently, the nomogram model has been established and proven to accurately predict the risk of VTE in multiple kinds of cancers, including lung cancer [22, 23], breast cancer [17], ovarian cancer [18, 19], lymphoma [20], and spinal metastasis tumor [21]. Cai et al. [22] studied a group of patients with stage IA NSCLC and established a nomogram for predicting the probability of postoperative VTE risk. Their prediction model contained variables including age, preoperative D-dimer, and intermuscular vein dilation, which is different from the predictive factors included in the nomogram model in our study. This result may be explained by the heterogeneity of the study population. Our study enrolled lung cancer patients with stage I-IV, including NSCLC and SCLC. Furthermore, another possible explanation for this is that some patients in this cohort treated by EGFR-TKIs, bevacizumab, and dexamethasone, which is not usually used in the stage IA NSCLC patients. Beside those factors, previous studies [22, 23] mainly focused on the postoperative VTE risk in lung cancer. A recently reported study [27] established a nomogram-based risk assessment model for venous thromboembolism, but the number of enrolled patients was relatively limited. Compared with previous models, our study developed a nomogram model based on a large number of patients with a more general representation for lung cancer VTE risk prediction.

The association between the use of corticosteroids and VTE has been investigated widely. Previous studies demonstrated that inhaled or oral corticosteroids significantly increased the risk of PE in patients with chronic inflammatory diseases [28, 29]. Dexamethasone, a kind of corticosteroids, is widely used in various clinical scenarios of patients with lung cancer, such as brain metastasis and inflammatory pain caused by bone metastasis. Wolpert et al. [30] studied the thrombosis risk in patients with brain metastasis. They found that the use of dexamethasone was confirmed to be independently associated with VTE (OR 2.27, 95% CI 1.5–4.5,* P* = 0.011). Consistent with previous findings, our result revealed that dexamethasone was a significant risk factor for VTE (OR 3.88, 95% CI 1.52–9.95, *P* = 0.005). A possible biological mechanism could explain the association between dexamethasone and the development of VTE. Brotman et al.’s study [31] has provided evidence that oral dexamethasone leads to a procoagulant state in healthy volunteers. In their study, dexamethasone 3 mg twice daily or placebo was given for five days to healthy male volunteers. They found that dexamethasone increased clotting factor levels VII, VIII, and XI and fibrinogen. Further investigations are needed to explore the mechanisms behind this finding.

Our results revealed that lung cancer patients who received EGFR-TKIs treatment have an odds ratio of VTE incidence of 4.71 (2.90–7.67, *P* < 0.01) compared with patients without EGFR-TKI treatment. A number of recent studies have reported similar results. Yang et al. [8] found that the odds ratio of VTE occurrence was significantly increased in patients with EGFR-TKI treatment relative to patients without the treatment. Lee et al.’s study [32] also demonstrated that the treatment with EGFR-TKI was associated with a 60% increased risk of VTE, which is consistent with our findings. There are several possible explanations for this result. Prior studies have verified the cross-talk between EGFR and VEGFR pathways [33, 34]. Activation of the EGFR pathway increases the production of tumor-derived VEGF that acts on endothelial cells in a paracrine manner to promote angiogenesis [34]. Inhibition of angiogenesis by both EGFR-TKIs and macromolecule anti-EGFR antibodies could lead to thrombosis occurrence [8, 35, 36]. Secondly, previous studies revealed that platelet activation could be triggered by EGFR-TKI treatment and low-dose aspirin treatment is effective on EGFR-TKI-induced skin rashes [37, 38]. The increase in platelet activation will lead to formation of platelet plugs and clots. Another possible explanation for this is that majority of patients received EGFR-TKI treatment in this cohort were staged III and IV with high PS score, all of which are dependent risk factor for VTE.

Bevacizumab is a recombinant humanized monoclonal antibody against VEGF and is widely used in the treatment of lung cancer, either in monotherapy or in combination with chemotherapy or immunotherapy [39, 40]. The impact of bevacizumab on VTE in cancer patients was widely investigated. Nalluri and colleagues’ meta-analysis [9] included a total of 7956 patients with a variety of advanced solid tumors to evaluate the association of bevacizumab with VTE. Their findings revealed that the incidence of all-grade VTE was 11.9% among patients receiving bevacizumab treatment. Bevacizumab significantly increased the risk of VTE with an RR of 1.33 (95% CI 1.13–1.56; *P* < 0.001) compared with controls. Consequently, several recent studies concluded similar results in various cancer types [41–45]. Consistent with previous reports, our results demonstrated that bevacizumab was also identified as an independent risk factor for VTE with an OR of 3.14 (95% CI 1.46–6.77; *P* = 0.003). Several mechanisms could explain this observation. Firstly, by inhibiting VEGF-induced endothelium regeneration, bevacizumab may expose subendothelial procoagulant phospholipids, which could result in thrombosis [46]. Secondly, the reduction of nitric oxide and prostacyclin resulting by bevacizumab was significantly predisposed to thromboembolic events [47]. Thirdly, the increased expression of proinflammatory cytokines may promote the inflammation of the vessel wall and initiate thrombosis in an intact vein [48, 49]. Additionally, the prolonged survival by bevacizumab treatment may be one of the causes of the increased risk of VTE.

A number of limitations need to be noted regarding the present study. First, all case enrolled in the current study was from a single center, which inevitably introduced bias and weakened the statistical power. Second, the current study has significant inherent limitations caused by the retrospective study design. Third, previous studies implied that driver gene alterations might impact the VTE occurrence in lung cancer patients, but the detailed information on driver genes was not accessed in this study. The association of driver genes with VTE could not be analyzed in our study. Consequently, in order to address the critical issues mentioned above, there is a strong need to develop a study with a well-designed prospective design, multi-centers, and large-size samples with detailed gene information in the future.

In conclusion, we established and validated a novel nomogram for predicting the risk of VTE in patients with lung cancer. The nomogram model could precisely estimate the VTE risk of individual lung cancer patients and identify high-risk patients who are in need of a specific anticoagulation treatment strategy.

## Data Availability

The raw data supporting the conclusion of this article will be made available by the authors, without undue reservation, to any qualified researcher.
